# The incidence of Coronavirus disease 2019 (COVID-19) among vaccinated healthcare workers (HCWs): evidence for protection from hospitalisation from an Indonesian cohort

**DOI:** 10.1016/j.lansea.2023.100146

**Published:** 2023-01-11

**Authors:** Zisis Kozlakidis

**Affiliations:** World Health Organization, International Agency for Research on Cancer, Lyon, France

Maintaining adequate protection for healthcare workers (HCWs) becomes an acute need during a pandemic, achieved through adherence to virus-transmission barrier measures, provision of personal protective equipment, and vaccines. Regarding the latter, HCWs occupy an exemplar position, communicating to the public the vaccination benefits, and dispelling hesitancy.^1^ The ‘imperative of vaccination’, as defined by *The Lancet*,^2^ introduced a dynamic scientific debate, and therefore stands equally as imperative to publish an evidence-base that removes ambiguity on vaccination benefits while recognising the complexity of data sets and corresponding contexts.

In this issue of *The Lancet Regional Health – Southeast Asia*, Soegiarto and colleagues^3^ report on a retrospective cohort of 2878 HCWs from two major hospitals in East Java, Indonesia, describing the vaccination impact on the incidence, hospitalisation, and characteristics of COVID-19 cases. Their study compares these parameters between the pre-vaccination (April 2020–January 2021) and post-vaccination (January–October 2021) periods, and between the first and second COVID-19 waves (peaking in December 2020 and June 2021 respectively).

The authors report that the cumulative incidence of COVID-19 infection among HCWs during the pre-vaccination period was 15.1%, (30-day incidence of 1.57%), and 22.6% (30-day incidence of 2.34%) post-vaccination. The rate of hospitalisation was significantly reduced from 23.5% (pre-vaccination) to 14.3% (post-vaccination). These results align with studies from elsewhere in Indonesia^4^ and globally.^5^ The study by Soegiarto and colleagues^3^ provide detailed evidence within the Indonesian context. For example, the increase in relative incidence post-vaccination can be explained by the dramatic infections increase in the general population by the delta variant; the waning of the immune protection, particularly for partially vaccinated individuals; and reduced immunological correspondence of the inactivated virus vaccine with the delta variant.^6^ Another important observation is the difference in the hospitalisation of HCWs, with the total number of hospitalised participants being higher post-vaccination (because the total number of infections was significantly higher), while the proportion of hospitalised subjects/total infected participants was markedly reduced. This is attributed to the milder COVID-19 symptoms and the vaccine protection, in line with similar studies globally,^7^ and the update in hospitalisation criteria within Indonesia, allowing low-risk patients to self-isolate at home.

During the COVID-19 pandemic, the wide disparity in health-system capacities in South-East Asia was reflected on the outcomes for COVID-19 management, spread, and mortality.^8^ Studies similar to the one by Soegiarto and colleagues^3^ are crucial, in providing the granular, contextual interpretation of the collected data, which can then inform future national health policies. Specifically in Indonesia, the combination of new variants within a highly populated nation put extreme stress on the healthcare system, necessitated a nation-wide, multi-sectoral mobilisation of healthcare and laboratory capacities,^9^ and a tenacious vaccination campaign. However, unanswered questions remain, for example the relative influence of contributing factors to the recorded COVID-19 incidence. Importantly, no viral sequencing data was obtained from the infected participants of this study, thus limiting any extrapolated correlation between circulating viral variants and hospitalisation incidence. As is true for many topics, hospitals and communities in different contexts have unique challenges and methods for overcoming those challenges fully or partially. These regional (East vs West Java) and national variations (across the Indonesian provinces) have not been discussed adequately, and thus limit our understanding and the extent to which these results can inform national and international health policies, though the national efforts for a standardised approach to the pandemic should be recognised. Additionally, the work underlines the need to protect vulnerable individuals with comorbidities, however the stratification of high-risk individuals requires further sharpening.

From an individual health perspective, the link between hypertension and hospitalisation is indeed interesting and could improve the understanding of the relative, individual disease burden. However, the analysis is limited from the unavailability of further structured data from HCWs (and a possible validation using comparable cohorts). From a population health perspective, this work highlights that the surveillance, understanding and eventual control of infectious diseases, requires a sustainable flow of reliable data. While numerous agencies and initiatives support the development of microbiology capacities in resource-restricted settings, delivering clinical and public health impact requires a national integrated model, combining clinical, laboratory and demographic information. The pandemic has afforded effective demonstrators; however, the systematic implementation of such a model remains perhaps a distant aspiration.

Conclusively, this study provides strong evidence for the protective nature of vaccinating HCWs within a representative South-East Asian healthcare setting. It demonstrates the risk of hospitalisation in HCWs, and supports the case for additional protective measures for those at risk of severe disease, including, but not limited to, booster vaccination doses, particularly at times when transmission rates are high or expected to rise.

## Contributors

ZK conceptualised and wrote the paper.

## Declaration of interests

None. Where authors are identified as personnel of the International Agency for Research on Cancer/WHO, the authors alone are responsible for the views expressed in this article and they do not necessarily represent the decisions, policy, or views of the International Agency for Research on Cancer/WHO.
